# Viscous damping of tremor using a wearable robot with an optimized mechanical metamaterial

**DOI:** 10.1017/wtc.2024.15

**Published:** 2024-12-10

**Authors:** Suhas Raghavendra Kulkarni, Dino Accoto, Domenico Campolo

**Affiliations:** 1 Robotics Research Centre, School of Mechanical and Aerospace Engineering, NTU, Singapore; 2 Department of Mechanical Engineering, Ghent and Aalst Campuses, KU Leuven, Ghent, Belgium

**Keywords:** metamaterial, wearable robot, tremor suppression, viscous damping

## Abstract

Pathological tremors can often be debilitating to activities of daily living and significantly affect the quality of life. Such tremulous movements are commonly observed in wrist flexion-extension (FE). To suppress this tremor we present a wearable robot (WR) with a customized mechanical metamaterial (MM) as the physical human-robot interface (pHRI). The MM is optimized to conform to the user’s wrist posture and follow the hand’s Cartesian trajectory. This is done to minimize the shear between the pHRI and the user’s skin and consequently improve wearability. This WR is then used to effect a viscous tremor suppression using the velocity of the user’s wrist FE. We present a model for the interaction between the WR and the user with which we develop the viscous damping approach for tremor. This is then evaluated in simulation and using a dedicated test bed. This tremor suppression approach demonstrates an attenuation of 20–30 dB at various tremulous frequencies resulting in significantly lower tremor amplitudes due to the viscous damping.

## 1. Introduction

A tremor is defined as an involuntary, rhythmic oscillatory movement of a body part (Bhatia et al., 2018). While tremulous motion can be observed in most people to a small degree, it is not enough to affect activities of daily living (ADLs). However, pathological conditions may lead to very severe tremulous movements, affecting the quality of life. Such pathological tremors – referred to as tremors in this paper – affects a significant portion of the general population (Louis et al., 1998). Tremor is observed to large extents in wrist flexion-extension (FE) in the 3–12 Hz frequency band with an acceleration amplitude of 13 rad/s^2^ (Rocon et al., 2004; Pigg et al., 2020). The most prevalent causes of tremor are (i) Parkinson’s disease (PD) and (ii) essential tremor (ET) (Benito-León et al., 2004, 2005). While ET affects nearly 5% of the population over 65 years old (Louis, 2005), PD affects an estimated 1% of the population over 60 years of age (Elias and Shah, 2014; Benito-Leon, 2018). However, the exact mechanism of the condition is not wholly understood (Bhatia et al., 2018). The common treatment modalities for patients suffering from such tremors are either surgical or pharmacological. While pharmacological interventions are commonly the first course of action, the efficacy of the treatments appears to diminish over time (Obeso et al., 2017) while also causing secondary effects due to the drugs (Koller and Vetere-Overfield, 1989; Rg et al., 2004). Surgical alternatives are invasive, present a higher risk, and may lead to secondary complications (Piasecki and Jefferson, 2004; Kleiner-Fisman et al., 2006; Dallapiazza et al., 2019).

As an alternative approach to managing tremulous movements, several wearable devices have been presented. As opposed to pharmacological or surgical interventions, wearable devices suppress the tremor by means of modifying the limb biomechanics. This is achieved by biomechanical loading applied to the limb through a wearable robot (WR) system. Another method to achieve the same biomechanical loading involves the use of functional electrical stimuation (FES) neuroprosthesis (Lora-Millan et al., 2021).

## 2. Associated challenges

Suppressing tremors using wearable devices adopts two primary approaches. The first approach includes solutions that rely solely on damping forces (Kotovsky and Rosen, 1998). However, such a passive device can be restrictive to the user. Another approach is to employ WRs by actively managing the tremulous motion (Matsumoto et al., 2013; Zhou et al., 2017; Awantha et al., 2020; Skaramagkas et al., 2020). While the tremor attenuation is seen to be effective from the presented results, these WRs are posed with challenges as any WR system that interfaces with a person. The wearable devices utilize either a rigid mechanism (Rocon et al., 2007; Herrnstadt and Menon, 2016) or soft elements such as cables (Zhou et al., 2017, 2018) and pneumatic actuators (Skaramagkas et al., 2020) to effect biomechanical loading and consequently suppress tremulous movements. While the rigid mechanisms enable effective force transmissions, minimizing the misalignment of the robot’s joints with that of the user is crucial (Schiele and Van Der Helm, 2006). Additionally, rigid devices often tend to be bulky limiting their wearability and effectiveness in assisting ADLs (Rocon et al., 2007). On the other hand, pneumatic actuators may bring with it a low bandwidth of operation (Wehner et al., 2012) and may cause discomfort due to shear because of a constantly varying area of contact (Davis et al., 2003). Furthermore, cable-driven devices often might need a careful choice of anchor points on the user’s limb to effect appropriate torque (Asbeck et al., 2014).

Alternatively, wearable devices for FES (Grimaldi et al., 2011; Bó et al., 2014) have shown promising results in suppressing tremulous movements. However major challenges in using FES techniques are first, the timing and control to accurately stimulate the muscles and second, the induced muscle fatigue (Maneski et al., 2011; Heo et al., 2015) which may reduce the effectiveness of the devices.

The challenges associated with the development of a tremor suppression system have motivated us to present a WR system utilizing an optimized mechanical metamaterial (MM) as the physical human-robot interface (pHRI) to suppress tremors in wrist FE. MMs are a class of architected structures and mechanisms that derive their properties from the arrangement of the constituent cells (Bertoldi et al., 2017). As such they offer a large design space that scales combinatorially with the number of cells. This design space can allow for the creation of mechanisms that enable precise and accurate motions like rigid mechanisms while also being conformal like the soft structures. Further utilizing an MM that is optimized to conform to a user’s joint posture and limb trajectory, we then develop a control model using the kinematic data of the user’s limb to effect tremor suppression with the wearable device.

In the following parts of this paper, we will provide an overview of the optimized MM and the wearable device in Section 3.1. Further a description and the modeling of the interaction between the WR with an MM and the user is provided in Section 3.2 along with the tremor suppression approach utilizing the user’s kinematic data. The results of the simulation and testing of the tremor suppression are reported in Section 4. The discussion and conclusion of the contributions of this paper are presented in Sections 5 and 9, respectively.

## 3. Methods

### 3.1. An overview of the customized wearable robot

**Figure 1.**
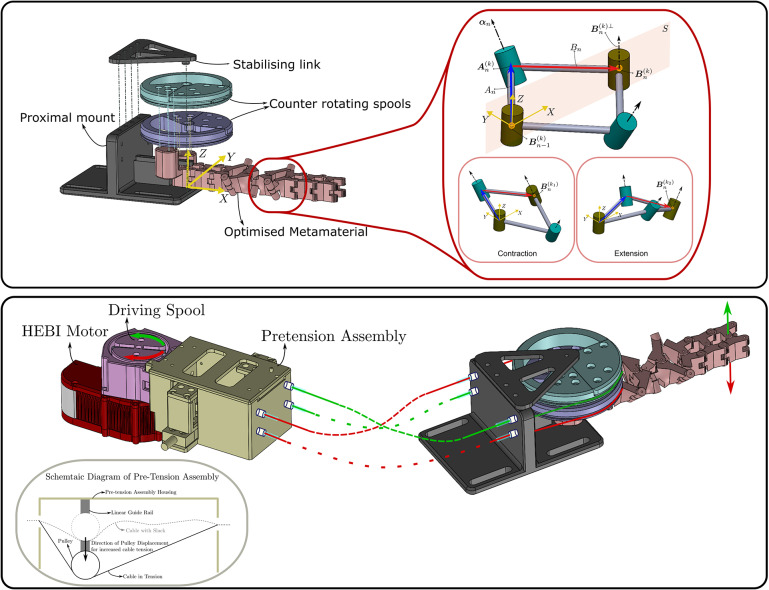
At the top, the optimized MM is assembled onto the proximal mount to constitute the pHRI. Also highlighted at the top is a unit cell of the MM where 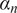 represents the orientation of the axis while the link length 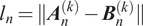 . Two pairs of red and green cables are routed through the spools for the actuation of the MM. When the green cables are in tension the MM bends upwards thus enabling wrist extension. When the red cables are in tension, the MM bends downwards enabling wrist flexion.

The physical structure of the WR is seen in Figure 1 has two components (i) the *pHRI* and (ii) a cable-driven actuation.

#### 3.1.1. Design of the mechanical metamaterial pHRI

The pHRI is composed of an optimized MM. The MM is composed of 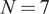 serially linked cells. Each cell is composed of four rigid links connected at four rotary joints thus constituting a four-bar mechanism as shown in Figure 1. Further, each cell of the MM can be either a bending cell or a planar cell depending on the orientation of the joint axes of the cell. Each cell in the MM is transformed from an initial flat state to another configuration by imparting a stimulus in the form of angular displacement 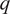 of one link in the cell. Furthermore, the behavior of the cell is dictated by two design variables of the cell which are (i) the length 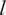 of the constituent links and (ii) the orientation of the cell axis in the initial flat state given as 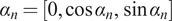 of the cell axis. The link lengths 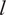 are equal for all the links of a cell and the cell axes are oriented symmetrically about a plane of symmetry (PoS) 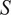 . Additionally, the cells are connected via ternary links that enable transmission of motion from one cell to the next. The MM has only a single degree of freedom (DOF) irrespective of the number of cells used to constitute the metamaterial.

Finally, the MM can be tuned to follow a desired behavior corresponding to a stimulus by altering the design variables of each cell of the MM. For an application in a WR to assist wrist FE the MM can be tuned to follow the Cartesian trajectory of a fixed point on the user’s hand and also conform to the posture of the user’s wrist. Such a customization can be achieved to minimize the shear between the pHRI and the user’s skin. We have implemented this in a gradient descent-based optimization approach and has already been described in Kulkarni et al. (2021, 2023). Using this approach the optimal parameters of an MM to conform to a specific user’s (29-year-old healthy 95th percentile male) wrist FE is described in Table 1.

**Table 1.** Optimized design parameters of an MM with seven cells to assist wrist FE

Cell	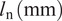	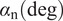
1	12.1	90
2	12.5	90
3	11.8	48.3
4	15.6	135.6
5	11.8	48.9
6	13.1	90
7	12.1	90

While we describe the MM designed to fit a specific user in this application, the kinematic model of the MM and the optimization approach developed to generate the design of the MM allow the creation of custom designs for each individual irrespective of their anthropometric class. We direct the reader to Kulkarni et al. (2023) for more details on the specific cost function used to generate the design of the MM as it is beyond the scope of this paper. This optimisation-based approach enables the development of pHRIs which not only minimize kinematic incompatibility but also minimized shear between the user’s skin and the pHRI for increased comfort.

#### 3.1.2. Actuation of the pHRI

To effect this actuation in a WR setup, we first need a proximal mount which will be used to

- Anchor the MM to the user’s hand.
- Provide anchor points for the actuating cables.

The MM is mounted to the user with the proximal mount which is designed to allow Velcro straps to anchor the device to the user’s hand. To provide an anchor for the cables actuating the MM, the vertical wall of the proximal mount has four holes placed at appropriate height to accommodate 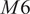 cable glands, which will secure the sheath of the Bowden cables to the proximal mount and the actuating cable can pass through to attach to the MM. The spool and cable clamp have been designed to clamp two cables, each of which can provide either 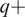 or 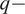 actuation to the MM resulting in a flexion or extension motion respectively. Since cables can only transmit tension, only one pair of these cables will enable actuation at any given point in time as shown in Figure 1. Further, the second spool is designed to be symmetric about 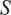 to the first spool. On the second spool, the cable enabling 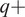 and 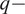 actuation is placed on the opposite side to that of the first spool, thus enabling symmetric actuation of the MM. The two spools are mounted co-axially using a dowel pin and bearings to enable frictionless counter-rotation.

Finally, the MM cells are designed such that the bottom surface of the links reflects the dimensions described in Table 1. Each link of the MM is 15 mm in height to allow for a low profile when worn by the user while still maintaining adequate strength to transmit the forces and torques for assistance. The links of the MM have been designed as parametric parts in Solidworks. This allows for rapid modifications to enable quick design changes. All the parts are produced using fused deposition modeling (FDM) which is an additive manufacturing (AM) process. The links are then assembled using dowel pins as the rotary joints. The wearable part of the actuated MM for supporting wrist FE weighs 350 g on the user’s limb.

To connect the four actuating cables to a motor, we have first developed a multi-level spool consisting of four independent channels to house the cables in. Since the actuating cables are only effective when transmitting tension, any slack in the system would hinder the desired operation of a device. For this reason, a pre-tensioning setup is included as shown in Figure 1. In this setup, pulleys are mounted on platforms which are in turn mounted on lead screws (X-axis feed screw, MISUMI SE Asia). The feed screw setup enables the translation of the pulleys in a direction perpendicular to the cables. The tension in the cable can thus be calibrated by adjusting the position of the feed screw. During assembly, the feed screw was driven using an electric drill to repeatably apply 8 Nm torque and thus tighten each cable to the same tension. The primary goal of this pre-tensioning setup was to ease assembly and remove any slack in the cables before the operation of the device. As such no specific tension goal was set.

For a cable-driven system, the torque transmitted can be determined as a function of the spool diameters at the driving and driven ends as(3.1)

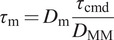

where 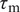 is the commanded motor torque, 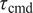 is the torque transmitted to the MM at the point of attachment of the driven spool, 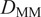 is the diameter of the driven spool and 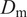 is the diameter of the spool attached to the motor. For the application of assisting wrist FE, the maximum torque at the wrist is 8 Nm (Morse et al., 2006; Yoshii et al., 2015). To accommodate this within a WR a HEBI X8-9 motor (continuous torque 8 Nm, peak torque 20 Nm) is chosen. Additionally, the diameter of the MM spool 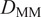 is chosen to be 80 mm and 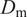 to be 48 mm thus providing a mechanical advantage of 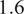 . Finally, the choice of the HEBI X8-9 motor is also driven by the fact that these motors are integrated modules (integrated torque sensing).

### 3.2. Control loop and suppression approach

**Figure 2.**
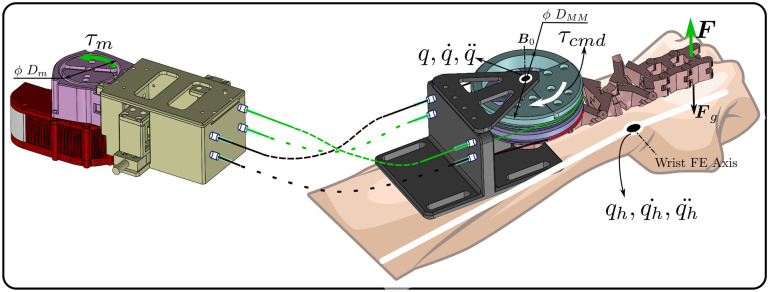
A 3D diagram of the wearable robot with the user’s hand indicating the torques and interaction forces.

The interaction forces and torques in the WR system can be visualized as seen in Figure 2. Let us first start with the model of the WR. The goal of this model is to capture the behavior of the robot corresponding to input torque. To describe such a system, we will utilize a 2nd order system. The equation of robot dynamics can be written as.
(3.2)

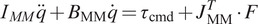

where 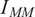 is the inertia of the actuated part of the WR, 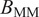 is the friction in the WR system primarily due to the friction of the Bowden cables. 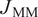 is the Jacobian of the WR relating the frame of the motor to the frame at the distal mounting point 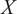 . 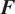 is the 3 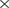 1 vector representing the two forces and a torque at the interface of the distal mount of the WR and the user’s hand. Also, 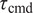 is the torque commanded to the motor.

Further, the user’s hand can also be modeled as a 2nd order system (Davidson and Charles, 2017) whose parameters are estimated based on the user’s anthropometric class from the sources (de Leva, 1996; Gomi and Osu, 1998; Halaki et al., 2006; Drake and Charles, 2014). As such the equation of the hand and wrist FE dynamics can be written as(3.3)

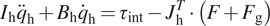

where 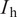 and 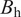 are the inertia and damping associated with the wrist FE. 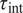 is the torque generated at the wrist due to voluntary movement by the user which can be estimated using kinematic and biomechanical parameters from Equation 3.3. 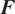 is the force vector at the distal mount of the WR due to the interaction of the WR with the user and 
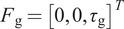
 is a posture-dependent parameter to enable gravity compensation. Additionally the centrifugal force terms have been neglected in Equations 3.2 and 3.3 since we are not dealing with very high velocities in our application.

**Figure 3.**
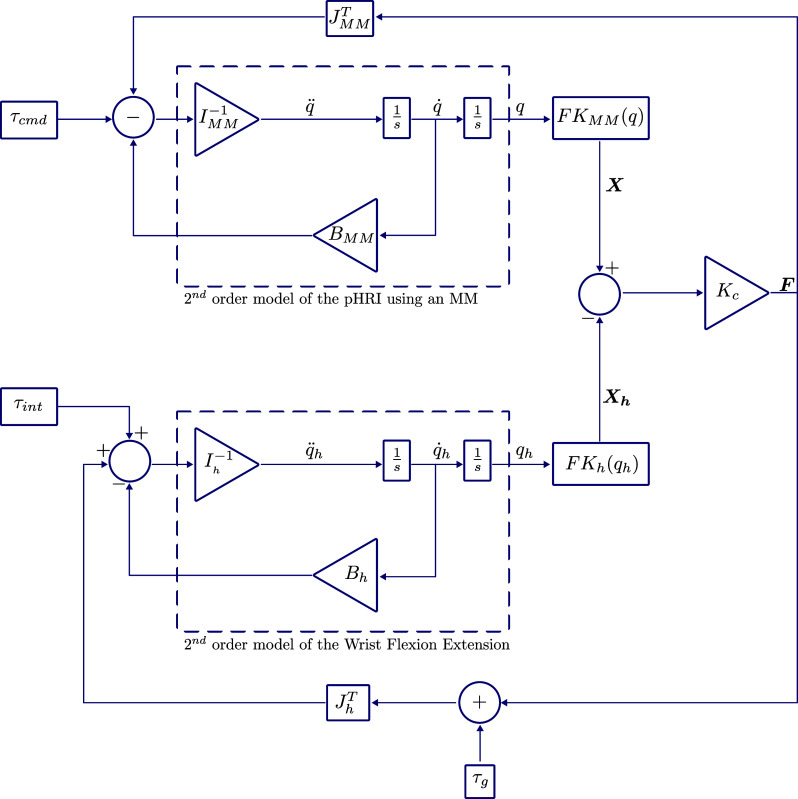
A dynamic model of the interaction between the WR using an optimised MM and the user. In the diagram, 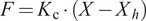 . Also 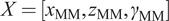 is the frame at the distal mounting point of the MM, 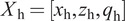 is the frame at the intended mounting point of the WR on the user’s hand and 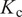 is the coupling stiffness between the WR and the user. 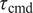 is the torque commanded to the motor and 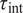 is the torque generated at the joint due to the user’s intent.

The interaction of the hand with the WR can be modeled using the mismatch between the frames of the hand and the distal mounting point of the WR. Thus the 2nd order model of the WR is coupled to the 2nd order model of the user’s hand (depicted in Figure 3) as(3.4)

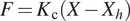


where 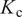 is a 3 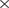 3 diagonal matrix denoting the coupling stiffness between the WR and the user. 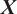 and 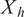 are the frames in 2D Cartesian space of the distal mounting point of the WR and the user’s hand, respectively. These frames can be given by(3.5)

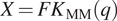


(3.6)

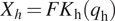

While the forward kinematics of the WR have been described in Kulkarni et al. (2021), the forward kinematics of the user’s wrist FE can be computed as a 1 DOF link.

To simulate the functioning and the interaction of the WR and the user, the values of various dynamic parameters such as inertia and damping are to be chosen.

The inertia parameter for the WR is estimated using the complete assembly in Solidworks to be 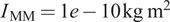 . The damping parameter however is estimated from the description provided in Xiloyannis (2019)) since the same cables and Bowden sheath are utilized in developing the cable-driven actuation of the WR. This parameter is chosen to be 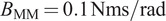 . The inertia and damping parameters for the wrist FE is gathered from the compilation in Davidson and Charles (2017). As such the inertia is chosen to be 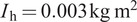 and the damping parameter is chosen to be 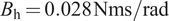 . Additionally, the gravity compensation torque 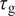 is introduced in the model as a simple specific posture-dependent torque to support the wearer in that posture as(3.7)

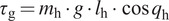

 where 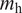 is the mass of the hand, *g* is the acceleration due to gravity and 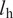 is the distance of the center of gravity of the hand from the wrist FE axis. For the purpose of this simulation, these values have been obtained from a computer aided design (CAD) model of a human forearm and hand with representative anthropometric dimensions. As such 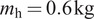 and 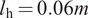 .

**Figure 4.**
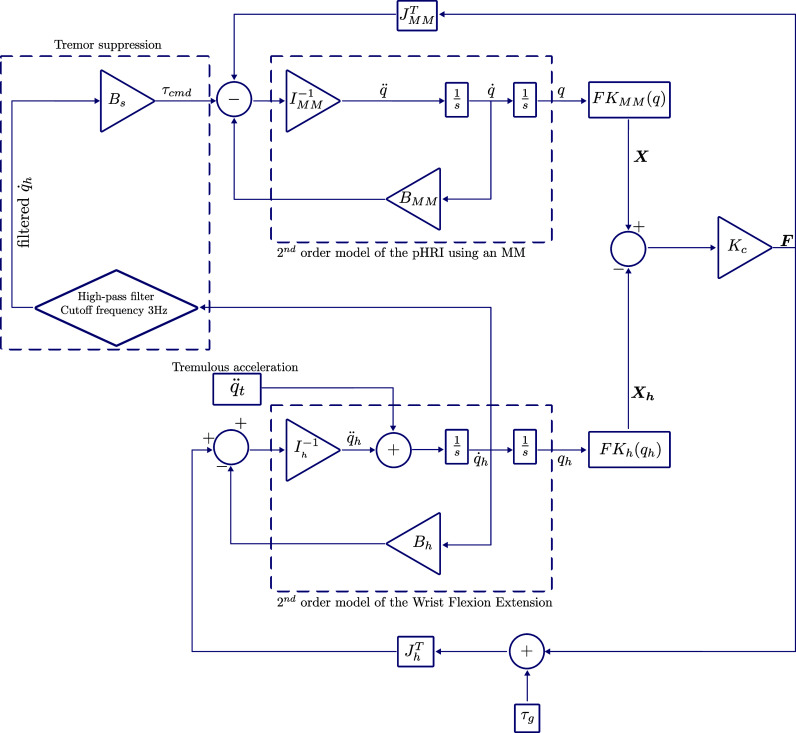
The implementation of the viscous damping approach for tremor suppression. This model has been built upon the previously described interaction model of the WR with the user.

Further, the coupling stiffness between the user’s hand and the WR is estimated to be 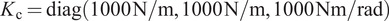 . This coupling stiffness is based on the stiffness of the nylon webbing used at the distal mounting point. The estimation is based on the assumption that all the load transferred to the user through the webbing is by means of longitudinal extension of the webbing. Using the estimated and chosen parameters a model is implemented in Simulink as shown in Figure 4.

### 3.3. Evaluation and testing of tremor suppression

#### 3.3.1. Simulation

Tremulous movements are composed of two components. First is the voluntary motion component and second is the tremulous component. These motions are seen to be in distinct frequency bands (Rocon et al., 2004). As such, tremulous movements due to Parkinson’s disease and essential tremors are consistently seen to have frequencies >3 Hz while voluntary motion is in the 0–3 Hz band. Further, the amplitude of tremulous acceleration is 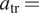 13 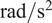 (Rocon et al., 2004). As such tremor is introduced into the simulation as(3.8)

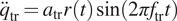

where 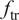 is the frequency of the tremulous movement and 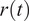 is a stochastic value between 0 and 1 generated at each time step using a Mersenne Twister Generator with the rand function in Matlab. This is to model the stochastic nature of tremor (Randall, 1973; Gantert et al., 1992).

As such to suppress only the tremor and not affect voluntary motions, we can apply a viscous damping torque to the joint based on the frequency of the motion.

To isolate the tremulous motion from the overall motion, the angular velocity of the user’s hand was high-pass filtered (2nd order Butterworth, cut-off frequency 3 Hz) and the tremor suppression torque was commanded using the filtered velocity as(3.9)

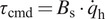

where 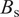 is the viscous damping coefficient chosen to be 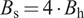 . This value has been chosen based on the sensitivity analysis presented in Davidson and Charles (2017). While higher 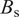 values will attenuate tremor more aggressively, the attenuation in the 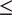 3 Hz range is also evident and this observation has restricted the choice of 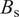 . Further, 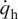 is the high-pass filtered velocity of the wrist in FE. Furthermore, the choice of 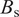 is primarily evaluated using a Bode plot of the controller to ensure that the bandwidth of the designed system spans the tremulous frequency range (3–12 Hz). This evaluation is carried out using the *Model Linearizer* tool in Simulink.

#### 3.3.2. Test setup

**Figure 5.**
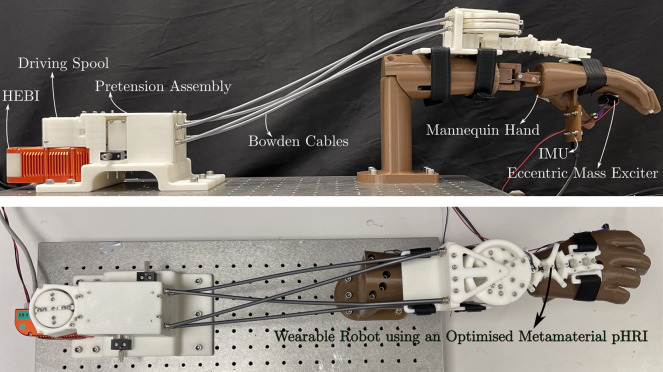
Test setup for evaluation of the tremor suppression approach using a WR with an optimized MM as the pHRI.

To evaluate the performance of the WR in suppressing the tremor, the setup as shown in Figure 5 is employed. The Mannequin hand is designed such that the various anthropometric dimensions represent the dimensions of the 95th percentile male as described by Tiley and Henry Dreyfuss Associates (2011). These dimensions are reflective of the specific user the MM has been optimized for.

An inertial measurement unit (IMU) is mounted on the hand of the mannequin such that the roll axis of the IMU is parallel to the radial-ulnar deviation (RU) axis of the wrist and the pitch axis to the FE axis of the wrist. The chosen IMU (SparkFun Razor 9DOF) is able to provide accelerometer and gyroscope data at 1 kHz sampling rate. As such the data is low-pass filtered at a cutoff frequency of 100 Hz to eliminate noise. Further, the IMU is also able to natively output Euler angles using the data from the accelerometer and the gyroscope. As such, the data from the IMU along with the data on the orientation of the HEBI motor (also provided natively from the motor) is sufficient to establish the posture of the wrist. This posture aids in providing the appropriate gravity compensation torque. Further, the gyroscope data provides angular velocity measures. As such the gyroscope output of the IMU which is given as a vector 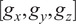 in the body-frame of the IMU, 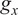 represents the angular velocity about the FE axis, and 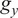 represents the angular velocity about the RU axis.

Further, an eccentric mass exciter (EME) is used to inject tremulous motion into the mannequin. The EME is composed of a low current DC motor with a link mounted at the shaft and a mass mounted at the end of the link asymmetrically with respect to (w.r.t) the shaft of the motor to act as the eccentric mass. The excitation force 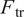 provided by the EME can be modeled (Rao, 2011) as(3.10)

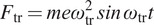

where 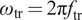 and 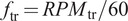 . The physical parameters of the EME are eccentric distance 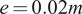 and eccentric mass 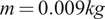 . The voltage–frequency relation of the EME is characterized experimentally using a tachometer and a DC power supply. A linear regression of this data is performed and the coefficients are slope 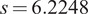 and intercept 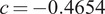 to relate the DC voltage to the tremulous frequency 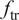 with the equation(3.11)

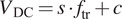



As such, the torque generated by the EME can be computed as(3.12)

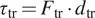

where 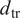 is the distance of the EME axis from the FE axis of the wrist. In the current setup 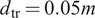 . Consequently, the simulated tremulous acceleration 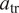 of the hand can be computed as(3.13)

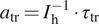



Thus the EME enables the generation of a tremulous acceleration amplitude of 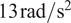 .

Such tremulous motion is injected into the mannequin hand at frequencies of 10, 8, 6, and 4 Hz by controlling the voltage. The tremulous motion caused due to the EME was identified and isolated by performing a high-pass filter with a cutoff frequency of 3 Hz using the Matlab function *highpass* on the data from the gyroscope 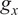 . The filtered velocity 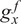 is used to command the viscous damping of the tremulous motion.

Further, the HEBI X8-9 motor driving the WR was controlled using the HEBI Matlab API. The data from the motor are sampled at a rate of 1 kHz and low-pass filtered at a cutoff frequency of 100 Hz to eliminate any noise. Additionally, a tremor suppression flag is set to either 0 or 1 based on a user’s input. When the flag is at 0, the tremor suppression does not occur and the WR functions only in gravity compensation mode. While the tremor suppression flag is set to 1, the wearable robot provides viscous damping using the filtered FE velocity 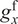 in Equation 9 where 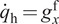 .

The test is performed starting at a tremulous excitation frequency of 10 Hz and stepped down to 4 Hz in steps of 2 Hz. Once the tremulous frequency of interest was achieved (by means of voltage control on the DC power supply of the motor according to Equation 3.11) the particular frequency was held for 10 s after which the tremulous frequency was changed to the next step. The WR was operated first in gravity compensation mode and the entire frequency band from 4 Hz to 10 Hz was traversed and the data was recorded using the IMU. Following this, the WR was switched to tremor suppression mode and the test was repeated.

## 4. Results

The primary metrics for the evaluation of tremor suppression devices are (Lora-Millan et al., 2021).

- Peak amplitude of the tremor.
- Root mean square (RMS) of the amplitude of tremor.
- Power spectral density (PSD).

These metrics provide insight into the nature of the interaction of the WR with the setup.

### 4.1. Simulation of tremor suppression

**Figure 6.**
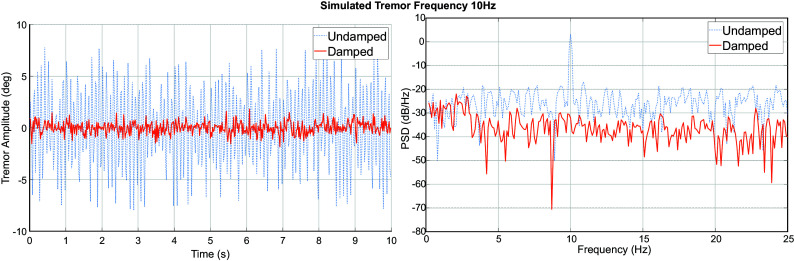
Simulation of 10 Hz tremulous motion in Simulink and the associated PSD.

From the results of the simulation of tremor at 10 Hz in Figure 6 it can be seen that the adopted approach of damping tremulous motion helps achieve a significant attenuation of tremor. Further, the results of this and other simulations carried out at different frequencies are summarized in Table 2.

**Table 2.** RMS and peak amplitudes of tremor in simulation with and without damping from the WR

Excitation frequency 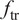 (Hz)	RMS amplitude (deg)		Peak amplitude (deg)	
	Undamped	Damped	Undamped	Damped
4	1.1	0.3	1.7	0.7
6	1.6	0.5	5.2	0.8
8	2.4	0.4	6.4	1.1
10	3.8	1.2	8.1	1.8

Further as seen from the power spectral density in Figure 6 the attenuation is observed to affect only 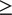 3 Hz while the movement below 3 Hz is transmitted without any effect.

### 4.2. Bench-test of tremor suppression

First a summary of the RMS and peak Table 3. The attenuation of the tremor in both peak and RMS metrics is evident from the results.

**Table 3.** RMS and peak amplitudes of tremor with and without damping from the WR in test

Excitation frequency 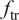 (Hz)	RMS amplitude (deg)		Peak amplitude (deg)	
	Undamped	Damped	Undamped	Damped
4	1.2	0.3	1.9	0.9
6	2.7	0.6	3.9	1.9
8	3.6	0.7	5.5	2.3
10	4.8	1.5	7.6	3.5

**Figure 7.**
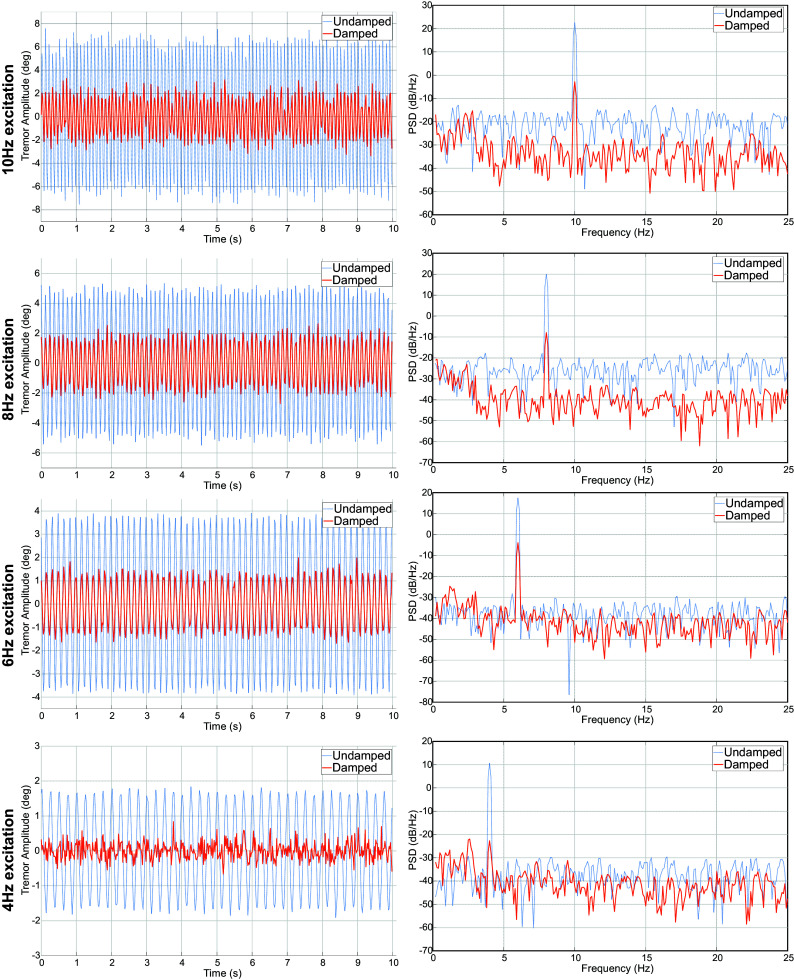
A comparative plot of tremulous motion of the mannequin hand at various frequencies with and without viscous damping tremor suppression.

Further, the PSD is presented in Figure 7 provides an insight into the nature of the interaction of the WR with the user. While the impact on the interaction in the ≤3 Hz band is minimal (≤1 dB), the attenuation of the tremor is in the range of 20 dB–30 dB depending on the tremor frequency 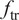 . Additionally, the impact of the viscous damping in the ≥3 Hz band is evident from the attenuation seen w.r.t the undamped PSD.

## 5. Discussion

In this paper, we have presented a method to model the interaction between a WR using an optimised MM as the pHRI and the user. Further, this model has been extended to simulate tremulous motion and a viscous tremor suppression approach has been implemented.

First, on the use of the optimized MM as the pHRI in the WR system. The MM is able to demonstrate complex trajectories that follow the user’s limb despite having a single DOF. This is possible due to the MM’s ability to bend and stretch/contract as it transforms from one state to the other based on the stimulus 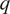 . This enables the MM to not only conform to the user’s joint posture but also match the strain of the user’s skin thereby minimizing the shear between the pHRI and the user. Furthermore, the developed methodology using a gradient descent-based optimization approach as detailed in our previous publication (Kulkarni et al., 2023) allows the MM to be customized to different users. This approach allows for mechanisms to be generated for each individual based on their anthropometric class. Consequently, the MM can embody the mapping between the trajectory of the user’s limb and the corresponding single stimulus ( 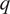 ) needed for the MM to match the user’s current joint state and this mapping is unique to each user. Additionally, the cable-based actuation of the WR allows for the heavier elements of the WR to be placed proximally (e.g., on the waist) allowing for the “worn” part of the WR to be as lightweight as possible. The 350 g weight of the WR mounted on the user’s hand in our case follows within the criterion used in Aubin et al. (2014) for the device weight to be ≤40% of the whole hand.

Next, the model of human joint motion as a second-order system is a commonly adopted approach (Pledgie et al., 2000; Rocon et al., 2004; Taheri et al., 2011). The second-order model is formulated using acceleration and velocity. These parameters are easily measured using sensors such as inertial measurement units (IMUs) which are composed of accelerometers and gyroscopes. The inertial and damping parameters of the optimized pHRI can be calculated in a similar manner to that presented in Tagliamonte et al. (2011). Following this, the characterization of the open-loop human–machine model can be undertaken. This can allow for the development of customized and individualized assistance protocols.

Further, the interaction of the WR with the user is through the coupling member (in this case the straps that bind the WR to the user’s hand) the coupling stiffness 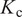 used in simulation is only an approximation since the estimation of true 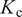 is not a trivial task. The properties of the material used along with the soft-tissue deformation play a significant role in determining the interaction between the pHRI and the user (Wolf et al., 2020). Additionally 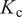 can be akin to a threshold function where if 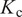 is too low, the assistance rendered is insufficient. In the case of this preliminary test and analysis, the choice of 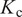 to effect tremor suppression provides a starting point for the development process. However, while applying the device and control strategy to patients the value of 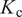 needs to be appropriately characterized within that environment to ensure fundamentally the safety of the patient and additionally the effectiveness of the WR. The development of a robust methodology to estimate biomechanical parameters ( 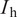 , 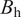 ) and the coupling stiffness ( 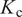 ) is a necessity.

The tremor suppression approach detailed in this work utilizing only the velocity of the wrist FE 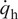 is a simple yet effective solution. As shown from the results, the attenuation of the tremor is in the range of 20 dB–30 dB depending on the frequency of the tremor. Furthermore, the choice of viscous damping coefficient 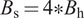 is driven primarily by the sensitivity analysis presented in Davidson and Charles (2017). While such an approach enables the demonstration of potential, a more robust methodology must be adopted to optimize the viscous damping coefficient 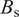 to the individual user and their tremor characteristics. Additionally, the usage of graded 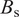 based on individual tremor characteristics as opposed to a single value can add to the effectiveness of this approach (Zahedi et al., 2021).

While the potential of such an approach is evident from the results presented, a true evaluation of the WR and the adopted control methodology is through real-world evaluations. As such conducting clinical evaluations for tremor suppression using this WR would provide a true measure of performance. Such clinical evaluation can include perspectives on the intrusiveness of the WR into the intentional motion and user comfort in addition to tremor suppression. Furthermore, sensing physiological signals such as electromyography (EMG) in addition to kinematic data from the IMU can provide an additional channel of information to estimate the user’s intention more accurately. As such, integration of this sensing modality into the WR control strategy during human-in-loop testing can enable better separation between tremulous movements and intentional movements.

## 6. Conclusion

In summary, the presented interaction model and the tremor suppression approach demonstrate the potential to warrant a real-world evaluation of the WR. The effectiveness of the WR in suppressing tremor simulated using an EME is evident from the presented results. Additionally, the interaction model as described in Figure 3 can be the first step in developing a multitude of control paradigms for the WR, and the tremor suppression approach serves as a demonstrator for the potential of the interaction model. Further, developing control models can also be done via computational approaches in which a first-level optimization of the MM is done to minimize shear and then a second optimization can be done for the specific control problem.

## Data Availability

The data that support the findings of this study are available from the corresponding author, DC, upon reasonable request.
